# Mechanistic Insights into the Stimulatory Effect of Melanogenesis of 4-Methylcoumarin Derivatives in B16F10 Melanoma Cells

**DOI:** 10.3390/ijms252212421

**Published:** 2024-11-19

**Authors:** Ye-Jin Lee, Chang-Gu Hyun

**Affiliations:** Department of Chemistry and Cosmetics, Jeju Inside Agency and Cosmetic Science Center, Jeju National University, Jeju 63243, Republic of Korea; yyyyejin615@gmail.com

**Keywords:** B16F10, GSK3β/β-catenin, MAPK, melanogenesis, 4-methylcoumarin, PI3K/Akt, primary skin irritation test

## Abstract

Vitiligo is a skin condition characterized by the loss of pigment, resulting in white patches on various parts of the body. It occurs when melanocytes, the cells that are responsible for producing skin pigment, are destroyed or stop functioning. This study aimed to investigate the melanogenic potential of various 4-methylcoumarin (4MC) derivatives, including 6-methoxy-4-methylcoumarin (6M-4MC), 7-methoxy-4-methylcoumarin (7M-4MC), 7-amino-4-methylcoumarin (7A-4MC), 6,7-dihydroxy-4-methylcoumarin (6,7DH-4MC), 7,8-dihydroxy-4-methylcoumarin (7,8DH-4MC), and 6,7-dimethoxy-4-methylcoumarin (6,7DM-4MC), in B16F10 melanoma cells. Our findings revealed that, while 4MC, 7A-4MC, 6,7DH-4MC, and 7,8DH-4MC did not exhibit any effect on melanin production, significant stimulation of melanogenesis was observed with 6M-4MC, 7M-4MC, and 6,7DM-4MC, with 6M-4MC demonstrating the most pronounced effect. 6M-4MC significantly stimulated melanin production and tyrosinase activity in a concentration-dependent manner in B16F10 cells. A Western blot analysis revealed that 6M-4MC increased the expression levels of microphthalmia-associated transcription factor (MITF), tyrosinase, tyrosinase-related protein-1 (TRP-1), and tyrosinase-related protein-2 (TRP-2). Further mechanistic studies showed that 6M-4MC inhibited extracellular signal-regulated kinase (ERK) and protein kinase B (AKT), which led to the upregulation of MITF and TRP proteins and subsequent activation of melanin synthesis. Additionally, 6M-4MC activated GSK3β phosphorylation, reduced β-catenin phosphorylation, and stimulated melanogenesis via the GSK3β/β-catenin pathway. Moreover, a primary skin irritation test was conducted on the upper backs of 32 healthy female volunteers to assess the potential irritation or sensitization from 6M-4MC when applied topically at concentrations of 50 µM and 100 µM. The test results showed no adverse effects on the skin. Collectively, these findings suggest that 6M-4MC may be a promising pigmentation stimulator for use in cosmetics and in the medical treatment of hypopigmentation disorders, particularly in the treatment of skin conditions such as vitiligo.

## 1. Introduction

Vitiligo is a chronic autoimmune skin disorder characterized by the localized loss of pigment, leading to the formation of white patches on the skin. It affects approximately 0.5% to 2% of the global population, making it a relatively common condition. The disease arises from the destruction or functional loss of melanocytes, the cells that are responsible for synthesizing melanin. When melanocytes are damaged, the affected areas of the skin become depigmented, resulting in the appearance of white patches. These depigmented spots predominantly occur on exposed areas of the body, such as the face, hands, feet, knees, and elbows, but they can develop anywhere on the body [1,2,3,4,5]. Although vitiligo does not typically cause physical discomfort such as pain or itching, its esthetic effects can lead to significant psychological and social stress for affected individuals, often resulting in mental health issues such as depression and anxiety [6,7].

Current treatments for vitiligo primarily involve the use of potent topical corticosteroids (TCSs), including betamethasone dipropionate, betamethasone valerate, clobetasol dipropionate, and fluticasone propionate [8]. These medications exert immunosuppressive effects that help inhibit the destruction of melanocytes and facilitate repigmentation. However, prolonged use of these agents can lead to serious side effects such as skin atrophy, pigmentary changes, telangiectasia, steroid dependence, and increased susceptibility to infections [9]. Due to these adverse effects, there is a pressing need to develop safer and more effective therapeutic options.

The primary goal of vitiligo treatment is to restore melanin production. Therefore, treatment strategies are aimed at either restoring the functionality of melanocytes or stimulating the synthesis of melanin. The current therapeutic approaches for vitiligo focus on preventing disease onset and promoting melanin synthesis. A significant correlation has been established between melanocytes, tyrosinase (TYR), and the manifestation of vitiligo [10,11]. In human skin, melanocytes are situated in the basal layer of the epidermis, where melanosomes within these cells serve as specific sites for melanin synthesis. The primary role of melanocytes is to produce melanin, a process mediated by melanocyte-specific enzymes that oxidize tyrosine. Melanin is stored within melanosomes and subsequently transferred to adjacent keratinocytes, thereby providing protection to the skin against ultraviolet (UV) radiation. Keratinocytes are essential for maintaining melanocyte homeostasis and melanin synthesis, forming functional and structural units within the skin [12,13]. The synthesis of melanin initiates with the conversion of L-tyrosine to L-DOPA, followed by the transformation into DOPA-quinone. These two reactions are catalyzed by the enzymatic activity of tyrosinase. Subsequently, DOPA-chrome is converted into 5,6-dihydroxyindole-2-carboxylic acid (DHICA) by TRP-2/DOPA-chrome tautomerase (TRP-2/DCT), which is then oxidized to indole-5,6-quinone-2-carboxylic acid by TRP-1/DHICA oxidase. These compounds ultimately polymerize into the brown-black eumelanin quinones [14,15,16]. To regulate this process, various synthetic and naturally derived functional components have been developed to modulate the activity of tyrosinase. Specific transcription factors, particularly the microphthalmia-associated transcription factor (MITF), play a crucial role in the signaling pathways of melanin synthesis. MITF upregulates the expression of TYR, thereby promoting melanin synthesis. As a result, MITF has emerged as a key factor in enhancing melanin production and functions as a central regulator of melanogenesis [17,18,19].

4-Methylcoumarin (4MC) is a derivative of coumarin, characterized by the substitution of a methyl group (-CH₃) at the 4-position of the basic coumarin structure. Its chemical formula is C₉H₈O₂, and coumarin typically exhibits a 1,2-benzopyrone structure, comprising two fused rings: a benzene ring and a pyrone ring. The pyrone ring contains an oxygen atom, which plays a crucial role in the compound’s biological activity. In 4MC, the methyl group is attached to the 4-position of the pyrone ring, functioning as an addition to one of the carbon atoms in the foundational coumarin structure [20,21]. This compound exhibits various biological activities and is regarded as an important substance in the study of synthetic and natural products. Notably, 4MC has demonstrated excellent antioxidant and radical scavenging properties in several experimental models. Since this compound is not metabolized by hepatic P450 monooxygenases, it does not form the mutagenic 3,4-coumarin epoxide, thereby helping prevent cellular damage and contributing to the prevention of various diseases associated with oxidative stress [22,23]. Moreover, 4MC derivatives display toxicity against various cancer cell lines, particularly inhibiting cell proliferation and inducing apoptosis. Studies have indicated that several derivatives exhibit significant inhibitory effects against the K562 (chronic myelogenous leukemia), LS180 (colon adenocarcinoma), and MCF-7 (breast adenocarcinoma) cell lines [21]. Furthermore, both 4MC and its derivatives exhibit anti-inflammatory properties, significantly inhibiting the production of nitric oxide (NO), thromboxane B2, prostaglandin E_2_ (PGE_2_), and tumor necrosis factor (TNF), positioning them as promising candidates for anti-inflammatory drug development [24]. Additionally, 4MC possesses antibacterial activity, particularly showing potential for development as an antitubercular agent [25,26]. Some studies have reported that 4MC derivatives exhibit neuroprotective effects, indicating beneficial potential in neurodegenerative diseases such as Alzheimer’s and Parkinson’s diseases. This compound effectively inhibits platelet aggregation, showing competitive antagonism at thromboxane A2 receptors and COX-1 inhibition, which may provide therapeutic options for patients who are resistant to conventional antiplatelet therapy [27,28]. Thus, 4MC can exhibit diverse pharmacological activities through structural modifications, serving as a vital foundational substance in the development of antioxidants, anti-inflammatory agents, anticancer drugs, antibacterial agents, and neuroprotective compounds. However, research regarding the relationship between 4MC derivatives and melanogenesis remains extremely limited.

**Figure 1:**
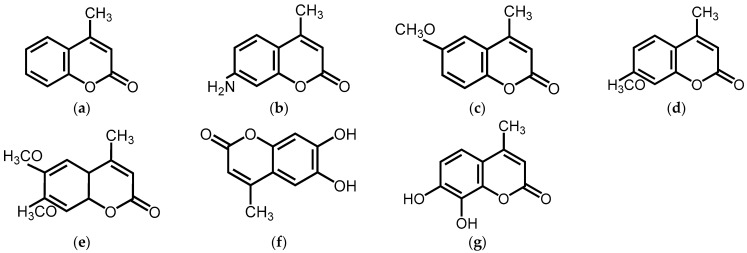
The chemical structures of 4-methylcoumarin (**a**) 4MC, 7-amino-4-methylcoumarin (**b**) 7A-4MC, 6-methoxy-4-methylcoumarin (**c**) 6M-4MC, 7-methoxy-4-methylcoumarin (**d**) 7M-4MC, 6,7-dimethoxy-4-methylcoumarin (**e**) 6,7DM-4MC, 6,7-dihydroxy-4-methylcoumarin (**f**) 6,7DH-4MC, and 7,8-dihydroxy-4-methylcoumarin (**g**) 7,8DH-4MC.

In previous studies, as part of our ongoing screening program aimed at discovering new cosmeceuticals and health supplements, we have identified several chalcones, psoralens, and coumarins that demonstrate anti-inflammatory properties and affect melanogenesis [29,30,31,32]. To further investigate these activities, this study focuses on 4MC and explores the impacts of specific structural modifications on its melanogenic activity. Specifically, we examined the structural characteristics and effects on melanin production of seven 4MC derivatives, including 6-methoxy-4-methylcoumarin (6M-4MC), 7-methoxy-4-methylcoumarin (7M-4MC), 7-amino-4-methylcoumarin (7A-4MC), 6,7-dihydroxy-4-methylcoumarin (6,7DH-4MC), 7,8-dihydroxy-4-methylcoumarin (7,8DH-4MC), and 6,7-dimethoxy-4-methylcoumarin (6,7DM-4MC), in B16F10 melanoma cells (Figure 1).

## 2. Results

### 2.1. Cell Viability and Melanin Content of 4MC Derivatives in B16F10 Cells

**Figure 2:**
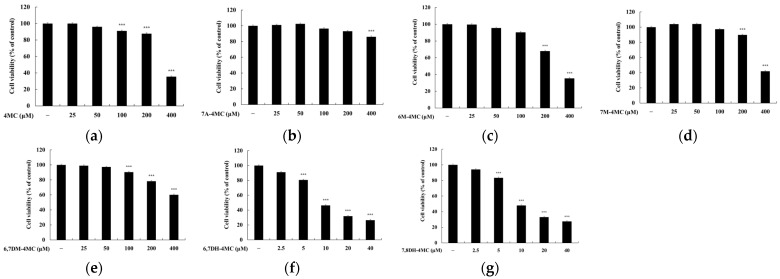
The effects of 4MC and its derivatives on the viability of B16F10 melanoma cells. The cells were treated with (**a**) 4MC (25 to 400 μM), (**b**) 7A-4MC (25 to 400 μM), (**c**) 6M-4MC (25 to 400 μM), (**d**) 7M-4MC (25 to 400 μM), (**e**) 6,7DM-4MC (25 to 400 μM), (**f**) 6,7DH-4MC (2.5 to 40 μM), and (**g**) 7,8DH-4MC (2.5 to 40 μM) for 72 h. Cell viability was expressed as a percentage relative to untreated cells. Results are expressed as the mean ± SD of three replicate experiments. *** *p* < 0.001 vs. the untreated control group.

To eliminate data errors during the investigation of the relationship between 4MC derivatives and melanogenesis, we assessed the cytotoxicity concentrations of the compounds in B16F10 cells using an MTT assay. B16F10 cells were treated with each compound at concentrations ranging from 25 to 400 μM and incubated for 72 h. Cell viability was considered unaffected if the survival rate was 90% or higher compared with the untreated control group. The experimental results showed that 4MC exhibited a cell viability of 87.75% at 200 μM and 90.95% at 100 μM. For 7A-4MC, the survival rates were 85.97% at 400 μM, 93.12% at 200 μM, and 96.51% at 100 μM. 7M-4MC displayed 89.75% cell viability at 200 μM and 97.29% at 100 μM. 6,7DM-4MC demonstrated 78.31% viability at 200 μM and 90.34% at 100 μM. In the case of 6,7DH-4MC, the cell survival rate was 84.98% at 5 μM and 96% at 2.5 μM. 7,8DH-4MC showed 83.42% viability at 5 μM and 94% at 2.5 μM. Finally, 6M-4MC demonstrated a survival rate of 67.95% at 200 μM and 90.38% at 100 μM. Given these results, subsequent experiments were conducted at a concentration of 100 μM for 4MC, 6M-4MC, 7M-4MC, and 6,7DM-4MC, while the remaining two compounds were tested at 2.5 μM (Figure 2).

**Figure 3:**
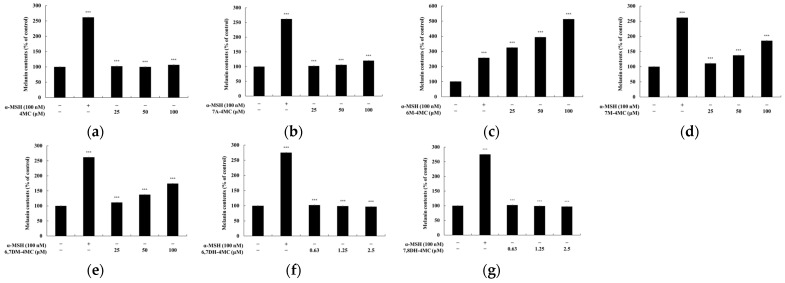
The effects of 4MC derivatives on the production of melanin in B16F10 melanoma cells. The cells were treated with (**a**) 4MC (25, 50, and 100 μM), (**b**) 7A-4MC (25, 50, and 100 μM), (**c**) 6M-4MC (25, 50, and 100 μM), (**d**) 7M-4MC (25, 50, and 100 μM), (**e**) 6,7DM-4MC (25, 50, and 100 μM), (**f**) 6,7DH-4MC (0.63, 1.25, and 2.5 μM), and (**g**) 7,8DH-4MC (0.63, 1.25, and 2.5 μM) for 72 h. The melanin content was expressed as a percentage relative to untreated cells. Results are expressed as the mean ± SD of three replicate experiments. *** *p* < 0.001 vs. the untreated control group.

In a preliminary study assessing the inhibitory or stimulatory effects of 4-methylcoumarin and its derivatives on melanin production in α-MSH-induced B16F10 cells, we found that most candidates did not inhibit melanogenesis. Instead, 6M-4MC was observed to significantly stimulate melanin production. Based on this finding, subsequent research focused on the melanogenesis-stimulating potential of 4-methylcoumarin and its derivatives. Specifically, B16F10 cells were treated with various concentrations of the compounds and incubated for 72 h, with α-MSH (100 nM) serving as the positive control. The melanin content was measured at non-cytotoxic concentrations. As shown in Figure 3, 4MC and 7A-4MC had no significant effect on melanogenesis at concentrations of 25, 50, and 100 μM. Similarly, 6,7DH-4MC and 7,8DH-4MC showed no effect at concentrations of 0.63, 1.25, and 2.5 μM. However, 6M-4MC, 7M-4MC, and 6,7DM-4MC significantly stimulated melanin production in a concentration-dependent manner at 25, 50, and 100 μM. Given these results, further experiments were conducted with 6M-4MC at concentrations below 100 μM, where no cytotoxicity was observed, to explore its strong melanogenic activity (Figure 3).

### 2.2. Effect of 6M-4MC on Tyrosinase Activity in B16F10 Cells

**Figure 4:**
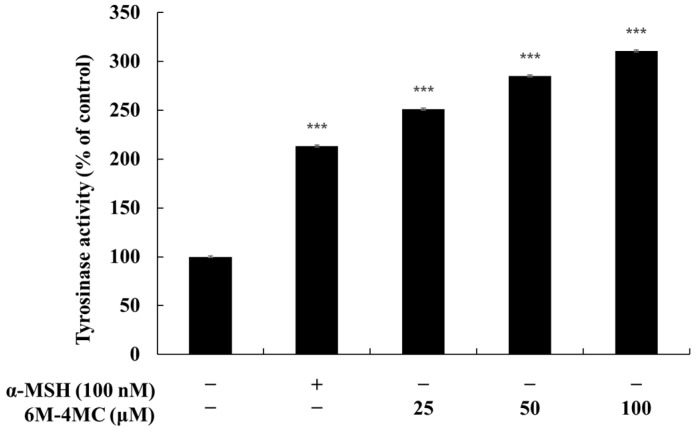
The effects of 6M-4MC on tyrosinase activity in B16F10 melanoma cells. The cells were treated with 6M-4MC (25, 50, and 100 μM) for 72 h. Tyrosinase activity was expressed as a percentage relative to untreated cells. Results are expressed as the mean ± SD of three replicate experiments. *** *p* < 0.001 vs. the untreated control group.

As mentioned in the introduction, tyrosinase plays a crucial role in melanogenesis, catalyzing the initial reaction that converts L-tyrosine to L-DOPA and then to dopachrome, thereby regulating the rate-limiting step of melanin synthesis. Therefore, the activity of tyrosinase significantly influences the quantity and type of melanin produced. In this study, we investigated the effects of 6M-4MC on tyrosinase activity in B16F10 cells. As shown in Figure 4, the cellular tyrosinase activity in the 6M-4MC-treated groups increased significantly in a concentration-dependent manner compared with the untreated control group, with a 210.6% increase at the maximum concentration of 100 μM relative to the untreated control. Additionally, when compared with the positive control, α-MSH (100 nM), 6M-4MC demonstrated a 79.6% increase in tyrosinase activity. These findings support the notion that 6M-4MC enhances tyrosinase activity in B16F10 cells, subsequently stimulating melanin production.

### 2.3. The Effect of 6M-4MC on the Expression of Melanogenic Enzymes and MITF

**Figure 5:**
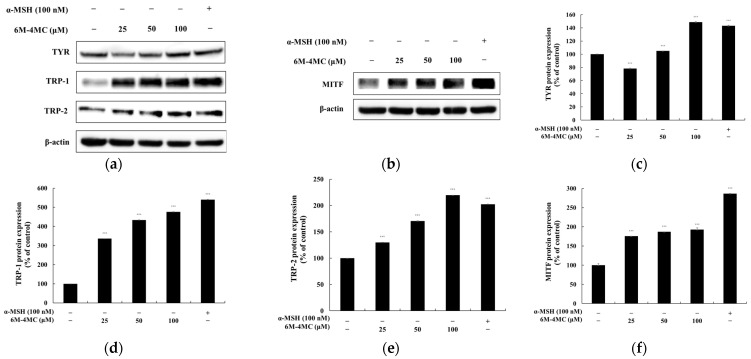
The effects of 6M-4MC on the expression of tyrosinase, TRP-1, TRP-2, and MitF proteins in B16F10 cells. Western blotting analysis illustrating the protein levels of tyrosinase, TRP-1, TRP-2, and MitF (**a**,**b**). Quantification of tyrosinase (**c**), TRP-1 (**d**), TRP-2 (**e**), and MitF (**f**) protein levels. The relative intensity of each protein band was analyzed using ImageJ software, and values were normalized to the corresponding loading control. Untreated cells were designated as 100%. Data are presented as the mean ± SD from at least three independent experiments. *** *p* < 0.001 compared with the untreated control group.

In the process of melanogenesis, tyrosinase serves as the key enzyme that converts L-tyrosine into melanin, while TRP-1 and TRP-2 play crucial roles in promoting melanin synthesis. The expression of these enzymes is primarily regulated by MITF, which enhances the expression of tyrosinase and TRP proteins, thereby contributing to the activation of melanogenesis. Therefore, MITF is significant as a major regulator of melanin synthesis. To evaluate the effects of 6M-4MC on the expression of melanogenic enzymes (tyrosinase, TRP-1, and TRP-2) and MITF, a Western blot experiment was conducted. As shown in Figure 5, the protein level levels of tyrosinase, TRP-1, and TRP-2 were significantly increased in the 6M-4MC-treated groups compared with the untreated control group. Specifically, at the highest concentration of 100 μM, the increases were 148.7%, 476.2%, and 219.8%, respectively, compared with the untreated group. Additionally, the expression of MITF was also increased by 6M-4MC in a concentration-dependent manner, with increases of 79.8%, 191.5%, and 194.5% at concentrations of 25, 50, and 100 μM, respectively. These results indicate that 6M-4MC enhances the expression of MITF, which in turn upregulates melanogenic enzymes and ultimately increases melanin biosynthesis.

### 2.4. The Effect of 6M-4MC on the Wnt/β-Catenin Signaling Pathway

**Figure 6:**
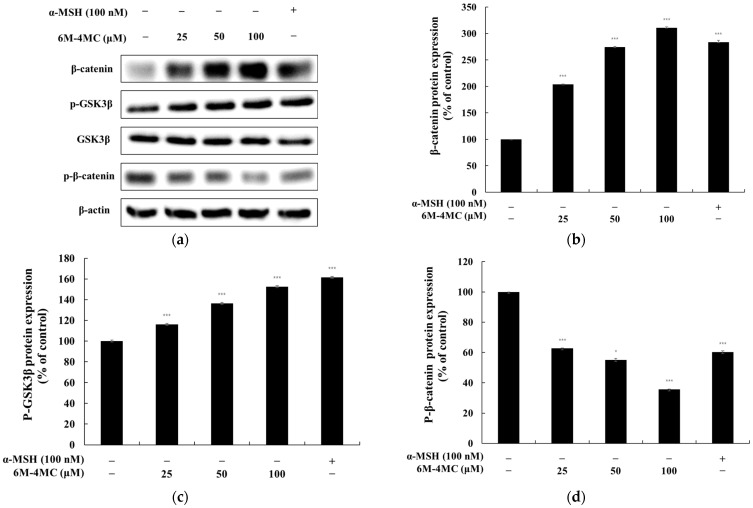
(**a**) Western blotting analysis demonstrating the protein levels of β-catenin, (**b**) β-catenin, (**c**) P-GSK3β/GSK3β, and (**d**) P-β-catenin. The relative intensity of each protein band was quantified using ImageJ software, and values were normalized to the corresponding loading control. Untreated cells were designated as 100%. Data are presented as the mean ± SD from at least three independent experiments. * *p* < 0.05, *** *p* < 0.001 compared with the untreated control group.

The Wnt signaling pathway plays a vital role in regulating the expression of MITF, and at the heart of this pathway lies the crucial enzyme GSK-3β, which is responsible for facilitating the ubiquitination and subsequent degradation of β-catenin; in this context, the phosphorylation of AKT, which occurs in the Wnt pathway, triggers the phosphorylation of GSK-3β, thereby inhibiting its activity, leading to the accumulation of β-catenin in the cytoplasm, which eventually translocates to the nucleus to enhance MITF expression, a process that ultimately results in the activation of TYR-family enzymes and stimulates melanin synthesis [33,34,35]. Therefore, we investigated whether 6M-4MC induces melanogenesis in B16F10 cells through the Wnt/β-catenin signaling pathway. The results showed that the 6M-4MC-treated group exhibited increased levels of P-GSK3β and β-catenin compared with the untreated control group. However, 6M-4MC inhibited the expression of P-β-catenin compared with the untreated group. These findings suggest that 6M-4MC increases melanogenesis through the Wnt/β-catenin signaling pathway (Figure 6).

### 2.5. The Effect of 6M-4MC on the AKT Signaling Pathway

**Figure 7:**
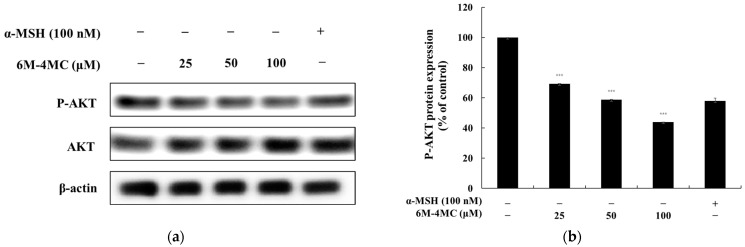
The levels of AKT and p-AKT were assessed using Western blotting. (**a**) Western blotting results illustrating the protein levels of AKT and p-AKT. (**b**) Quantification of the AKT and p-AKT protein levels. The relative intensity of the protein band was analyzed using ImageJ software, and values were normalized to the corresponding loading control. Untreated cells were designated as 100%. Data are presented as the mean ± SD from at least three independent experiments. *** *p* < 0.001 compared with the untreated control group.

The AKT signaling pathway has been reported to play a role in the regulation of melanin synthesis, with phosphorylated AKT inhibiting melanin production [36,37,38]. Consequently, we investigated whether 6M-4MC induces melanogenesis through the AKT signaling pathway in B16F10 cells. The results demonstrated that treatment with 6M-4MC significantly decreased AKT phosphorylation. These findings indicate that 6M-4MC promotes melanogenesis by reducing the phosphorylation of AKT within the AKT signaling pathway (Figure 7).

### 2.6. The Effect of 6M-4MC on the MAPK Signaling Pathway

**Figure 8:**
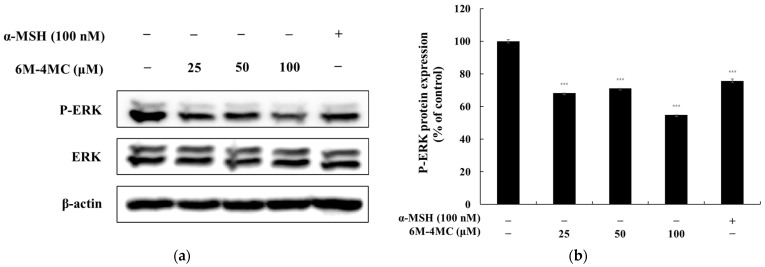
16F10 cells were treated with 6M-4MC for 4 h and then harvested. The expression of ERK was assessed using Western blotting. (**a**) Western blotting results illustrating the protein levels of ERK. (**b**) Quantification of ERK protein level. The relative intensity of the protein band was analyzed using ImageJ software, and values were normalized to the corresponding loading control. Untreated cells were designated as 100%. Data are presented as the mean ± SD from at least three independent experiments. *** *p* < 0.001 compared with the untreated control group.

The MAPK pathway is a major signaling cascade that regulates cell growth, differentiation, survival, and stress responses, comprising various protein kinases, including ERK, JNK, and p38 MAPK. This pathway plays a crucial role in melanogenesis, particularly through the phosphorylation of ERK, which regulates the expression of MITF. Consequently, MITF promotes the expression of melanogenic enzymes such as tyrosinase, TRP-1, and TRP-2, thereby increasing melanin synthesis [36,37,38]. Therefore, we investigated the effect of 6M-4MC on MAPK phosphorylation in B16F10 cells. As shown in Figure 8, at the highest concentration of 100 μM, 6M-4MC was found to decrease ERK phosphorylation by 54.8% in B16F10 cells. These results confirm that the activation of melanogenesis by 6M-4MC involves the MAPK signaling pathway.

### 2.7. 6M-4MC Is Safe for Human Skin

Human skin needs protection from environmental factors and chemicals in cosmetics and pharmaceuticals, especially in vulnerable groups such as children. Therefore, evaluating the potential of cosmetic ingredients to cause acute skin irritation is crucial. A skin irritation test was conducted to assess the effects of topical application of 6M-4MC at concentrations of 50 μM and 100 μM dissolved in squalene. Following the PCPC guidelines and Dermapro Inc. procedures, 32 women (mean age 43.03 ± 5.17 years) participated in a patch test where 20 μL of 6M-4MC was applied to clean areas of their backs for 24 h. Skin evaluations were performed 20 min and 24 h after patch removal. The results indicated that 6M-4MC has hypoallergenic properties in terms of primary skin irritation (Table 1).

## 3. Discussion

Coumarins derived from biological sources represent an important class of bioactive molecules with broad pharmacological applications, including anti-inflammatory, anticancer, and antimicrobial activities [39,40,41,42]. Their structural versatility, combined with advanced synthetic organic chemistry techniques, allows for the optimization of these natural scaffolds into highly potent lead compounds. This synergy between natural product chemistry and synthetic methodologies is pivotal in pharmaceutical innovation, enabling the development of novel therapeutics targeting various disease pathways [43,44]. Among these, 4MC, a naturally occurring compound found in both plants and fungi, stands out for its significant biological activity, particularly its antioxidant, anti-inflammatory, antimicrobial, and anticancer properties. In recent studies, small coumarin molecules have proven to be robust starting points for drug development, maintaining their appeal in therapeutic research [22,23,24,25,26,27,28].

Our laboratory has leveraged multiple assay platforms to demonstrate the functional efficacy of chalcones, psoralens, and coumarins in modulating inflammatory responses, obesity, and melanogenesis, offering promising insights into their broader application in drug discovery and development [29,30,31,32]. This study investigated 4MC and its derivatives, including 7A-4MC, 6M-4MC, 7M-4MC, 6,7DM-4MC, 6,7DH-4MC, and 7,8DH-4MC, in relation to melanin production. The results indicated that 4MC, 7A-4MC, 6,7DH-4MC, and 7,8DH-4MC did not exhibit any melanin production or inhibition effects. In contrast, 6M-4MC, 7M-4MC, and 6,7DM-4MC significantly activated melanin content and intracellular tyrosinase activity. Notably, 6M-4MC was found to strongly stimulate melanin production in mouse B16F10 melanoma cells compared with the other derivatives.

In terms of structure–activity relationships, the findings for 6M-4MC, 7M-4MC, and 6,7DM-4MC present intriguing insights into their effects on melanogenesis. Although these compounds share structural similarities, they exhibit significantly different degrees of melanin production activation. At a concentration of 100 µM, 6M-4MC led to a 512.6% increase in melanin content, while the use of 7M-4MC resulted in a 185.4% increase. However, 6,7DM-4MC, which features methoxy groups at both the C-6 and C-7 positions, only maintained a 174.4% increase, indicating no synergistic effect. One possible explanation is steric hindrance. In 6,7DM-4MC, the methoxy groups at C-6 and C-7 may cause steric interference, hindering access to melanogenic enzymes’ allosteric sites or transcription factors involved in signaling pathways [45]. This limitation could reduce the number of enzyme–substrate interactions, whereas 6M-4MC and 7M-4MC may avoid such steric hindrance, allowing for more effective binding with the enzyme. A second factor may involve the electron-donating properties of the methoxy groups, which influence the electron distribution across the molecule [46,47]. For 6M-4MC and 7M-4MC, the electron density may enhance interactions with enzymes, while 6,7DM-4MC may experience inhibitory effects from electron distribution alterations. Molecular flexibility is another aspect to consider [48,49]. 6M-4MC and 7M-4MC might have greater conformational flexibility, allowing for optimal binding orientations with the enzyme, whereas the more constrained ring structure of 6,7DM-4MC could limit its ability to orient itself for effective binding. Finally, hydrophobic interactions could also play a role in enzyme–ligand binding [50,51]. The methoxy groups at both C-6 and C-7 in 6,7DM-4MC might impede effective hydrophobic interactions with the enzyme’s active site. In summary, despite structural similarities, the differences in activation levels highlight how substituent positioning and properties critically influence enzyme interactions, suggesting the need for further investigation into these subtle structural variations.

In this study, 6M-4MC was shown to activate melanin production in B16F10 melanoma cells by upregulating tyrosinase, TRP-1, and TRP-2, indicating that 6M-4MC may stimulate intracellular tyrosinase activity by enhancing the expression of these melanogenesis-related enzymes (Figure 5). This aligns with prior findings that methoxylated natural products are generally more effective at activating rather than inhibiting melanogenesis [52,53]. Despite previous reports that coumarin compounds promote melanin synthesis, further exploration of coumarin derivatives remains scientifically valuable. First, modifications to the basic structure of 4-methylcoumarin can alter its effects on the melanogenesis pathway, with different substituents (e.g., methoxy or amino groups) modulating the activation or inhibition of key enzymes such as tyrosinase. This structural versatility may enable the development of optimized derivatives with enhanced efficacy or reduced side effects. In this study, while compounds such as 4MC, 7A-4MC, and 7,8DH-4MC did not affect melanin production, 6M-4MC, 7M-4MC, and 6,7DM-4MC demonstrated significant melanin content increases and intracellular tyrosinase activity. Furthermore, specific 4-methylcoumarin derivatives could exhibit stronger or more selective activity than the parent compound, due to additional substituents that reinforce enzyme interactions, even at lower concentrations. Also, while high doses of 4MC may cause skin irritation, studying derivatives allows for the discovery of formulations with high bioavailability and safety. Compounds with stable molecular structures that effectively induce melanin synthesis may be particularly useful for treatments like vitiligo or in the development of cosmetics designed to enhance tanning effects or provide a natural bronze tone to the skin. Additionally, new derivatives can reveal alternative mechanisms or pathways in melanogenesis, potentially regulating melanin production more comprehensively. For example, specific derivatives may interact with proteins in oxidative stress pathways or other signaling molecules in melanin synthesis, thus overcoming the limitations of existing compounds [54,55,56]. Therefore, research on 4MC derivatives is scientifically significant, as it not only complements existing coumarin-based studies but also offers promising, safer alternatives for treating hypopigmentation conditions, including vitiligo.

The study of signaling mechanisms that are involved in melanogenesis is crucial for the development of vitiligo treatments. Understanding the major pathways and proteins that regulate melanin synthesis is essential for creating effective therapeutics. Key signaling pathways involved in melanin synthesis include the Wnt/β-catenin, PI3K/Akt, PKA, and MAPK pathways [33,34,35,36,37,38]. These pathways influence the transcription factor MITF, promoting the expression and activity of melanin synthesis enzymes such as tyrosinase, TRP-1, and TRP-2. Given that vitiligo results from the loss of melanocytes, it is important to develop targeted therapies that can induce the proliferation of melanocytes or maintain their activity by modulating these signaling pathways. For example, the Wnt signaling pathway plays a significant role in the proliferation and survival of melanocytes, and drugs that activate this pathway could potentially be effective in treating vitiligo. Targeting specific signaling pathways can also help reduce the side effects that might arise from non-selective drug actions. This is particularly critical in the development of safe and effective treatments for sensitive skin. Therefore, understanding the signaling mechanisms related to melanin production in vitiligo research provides a scientific basis for enhancing the therapeutic efficacy, minimizing side effects, and developing personalized treatment strategies.

The Wnt/β-catenin signaling pathway consists of GSK-3β and β-catenin proteins, with GSK-3β being a constitutively active kinase that is phosphorylated by various kinases, including Akt and PKA. Recent studies indicate that this pathway is closely linked to melanogenesis, as β-catenin translocates from the cytoplasm to the nucleus, enhancing the transcription of MITF and subsequently increasing its expression. This interaction promotes the expression and activity of melanogenic enzymes, which are critical for melanin synthesis [33,34,35].

Our research demonstrated that 6M-4MC enhances the phosphorylation of GSK-3β, leading to increased accumulation of β-catenin in the cytoplasm. Consequently, this results in the overexpression of MITF, ultimately promoting melanin biosynthesis. Therefore, the melanin production mediated by 6M-4MC appears to be triggered via the Wnt pathway, which aligns with existing studies highlighting the role of GSK-3β and β-catenin activation in melanogenesis. This finding underscores the importance of the Wnt/β-catenin pathway as a significant mechanism regulating melanin synthesis. The PI3K/Akt signaling pathway plays a crucial role in the phosphorylation and regulation of MITF. The activation of the PI3K/Akt pathway enhances the phosphorylation of MITF, thereby increasing its transcriptional activity and stability. This, in turn, promotes the expression of enzymes associated with melanin synthesis, such as tyrosinase, contributing to melanin production. This relationship underscores the significance of the PI3K/Akt signaling pathway in regulating MITF activity [36,37,38]. Our data demonstrate that, in B16F10 cells, the levels of p-Akt are downregulated in response to 6M-4MC-induced melanin production. This finding suggests that the PI3K/Akt pathway may play a role in stimulating the melanin synthesis prompted by 6M-4MC. Further investigations into the specific mechanisms of this interaction could provide valuable insights into targeted therapies for pigmentation disorders. The MAPK signaling pathway is well established as playing a crucial role in the activation of MITF during the melanin production process. Notably, the inhibition of ERK signaling has been reported to increase tyrosinase activity, thereby promoting pigmentation [36,37,38]. In this study, we investigated whether 6M-4MC induces ERK inhibition in B16F10 cells. As shown in Figure 8, treatment with 6M-4MC resulted in a significant decrease in the phosphorylation level of ERK after 4 h. These findings suggest that 6M-4MC may induce melanin production in B16F10 cells by inhibiting ERK phosphorylation. Furthermore, this research indicates that the melanin formation induced by 6M-4MC and the expression of tyrosinase and MITF in B16F10 cells are functionally regulated by ERK MAPK. Collectively, our results imply that the activation of MITF through the Akt/GSK3β/β-catenin and MAPK pathways mediates the melanin-enhancing effects of 6M-4MC.

Skin irritation testing is essential in the development of topical products. These tests evaluate the safety of formulations and help identify potential side effects, which is critical for ensuring consumer safety. Protecting the skin from harmful effects is of the utmost importance. Additionally, regulatory agencies often require such assessments as part of their approval process for topical products. Therefore, we conducted a skin irritation test on 32 subjects for the topical application of 6M-4MC, following OECD guidelines. As presented in Table 1, our results confirmed the skin safety of 6M-4MC.

In summary, the discovery of effective natural compounds that promote melanin synthesis could significantly help control non-genetic pigmentation disorders and reduce side effects associated with chemical treatments, while also being applicable in cosmetics for safe tanning without UV exposure. Therefore, our study suggests that the candidate compound 6M-4MC is a promising pigment-stimulating agent that could serve as an effective strategy for addressing hypopigmentation issues and for developing tanning cosmetics. However, this study has certain limitations. The B16F10 mouse melanoma cell line is widely used in numerous studies related to melanin formation, and we employed the same cell line in our experiments. While 6M-4MC demonstrated melanin production effects at the cellular level, these results may not always correlate with those observed in human melanocytes or clinical settings. Therefore, to translate these findings into practical applications, it will be essential to determine the therapeutic efficacy of 6M-4MC for treating human hypopigmentation disorders through studies involving human melanocytes or clinical trials.

## 4. Materials and Methods

### 4.1. Chemicals and Antibodies

4-Methylcoumarin (4MC), 7-amino-4-methylcoumarin (7A-4MC), 7-methoxy-4-methylcoumarin (7M-4MC), 6,7-dimethoxy-4-methylcoumarin (6,7DM-4MC), 6,7-dihydroxy-4-methylcoumarin (6,7DH-4MC), 7,8-dihydroxy-4-methylcoumarin (7,8DH-4MC), and 6-methoxy-4-methylcoumarin (6M-4MC) were purchased from Tokyo Chemical Industry (Chuo-ku, Tokyo, Japan). Dulbecco’s Modified Eagle’s Medium (DMEM), penicillin–streptomycin (10,000 U/mL), fetal bovine serum (FBS) used for cell culture, and the BCA protein assay kit for protein quantification were obtained from Thermo Fisher Scientific (Waltham, MA, USA). The 3-(4,5-dimethylthiazol-2-yl)-2,5-diphenyltetrazolium bromide (MTT) used for the cell viability measurement, the protease inhibitor cocktail used for cell lysis, the α-melanocyte-stimulating hormone (α-MSH) used as a control, arbutin, L-3,4-dihydroxyphenylalanine (L-DOPA), sodium hydroxide (NaOH), sodium phosphate monobasic, sodium phosphate dibasic, and the 2-mercaptoethanol used for cell experiments were purchased from Sigma-Aldrich (St. Louis, MO, USA). The 2x Laemmli sample buffer, 10% Tween 20, and nonionic detergent for Western blot sample preparation were acquired from Bio-Rad (Hercules, CA, USA). Skim milk for membrane blocking was obtained from BD Difco (Sparks, MD, USA), and bovine serum albumin was sourced from Bovostar (Bovogen, Melbourne, Australia). Dimethyl sulfoxide (DMSO), radioimmunoprecipitation assay (RIPA) buffer, 20x TBS buffer (pH 7.6) for preparing 1x TBST, 10x Tris-glycine buffer, and phosphate-buffered saline (PBS) were purchased from Biosesang (Seongnam, Gyeonggi-do, Republic of Korea). The primary antibodies used were tyrosinase (SC-20035), TRP-1 (SC-166857), TRP-2 (SC-74439), β-actin (C4) (C-47778_s), and MITF (SC-71588), all purchased from Santa Cruz Biotechnology (Dallas, TX, USA). Secondary antibodies included p-β-catenin (9561S), β-catenin (25362S), p-GSK-3β (9322S), and GSK-3β (5676S) from Santa Cruz Biotechnology, as well as p-ERK (9102S), ERK (9101S), p-p38 (9211S), p38 (9212S), p-AKT (9271S), AKT (9272S), anti-rabbit IgG HRP-linked antibody (7074S), and anti-mouse IgG HRP-linked antibody (7076S) from Cell Signaling Technology (Danvers, MA, USA).

### 4.2. Cell Culture

The B16F10 mouse melanoma cells used in this experiment were purchased from ATCC (The Global Bioresource Center, Manassas, VA, USA) and incubated in DMEM containing 10% FBS and 1% penicillin–streptomycin at 37 °C, 5% CO_2_.

### 4.3. Cell Viability

The B16F10 cells were seeded in 24-well plates at 1.5 × 10^4^ cells/well and incubated for 24 h. Then, samples were treated in duplicate and incubated for 72 h in an incubator at 37 °C and 5% CO_2_. After the reaction, all the treated medium was removed, and the samples were treated with 500 μL per well of MTT reagent at a concentration of 0.2 mg/mL and incubated for 4 h in an incubator at 37 °C, 5% CO_2_. All medium was removed, and formazan crystals adhering to the bottom of the plate were dissolved using DMSO at 37 °C for 20 min. The lysate was transferred in 200 μL aliquots to a 96-well plate, and the absorbance was measured at 540 nm using a microplate reader (Epoch, BioTek, Winooski, CA, USA).

### 4.4. Measurement of Melanin Contents

The B16F10 cells were seeded at a density of 8.0 × 10^4^ cells in 60 mm dishes and incubated with various concentrations of samples and α-MSH (100 nM) for 72 h in a 5% CO₂ incubator at 37 °C. After incubation, the supernatant was carefully removed, and the cells were washed twice with cold 1× PBS. Then, 200 μL of lysis buffer (RIPA buffer containing 1% protease inhibitor cocktail, 100:1) was added per plate, and the cells were lysed by means of incubation at 4 °C for 30 min. The lysed cells were scraped from the plates using a cell scraper (SPL Life Sciences, Pocheon, Gyeonggi-do, Republic of Korea), transferred to a 1.5 mL microcentrifuge tube, and centrifuged at 15,000 rpm for 30 min at −8 °C. The resulting pellet was dissolved by adding 200 μL of 1N NaOH containing 10% DMSO, followed by incubation at 80 °C for 20 min. The solution was then transferred to a 96-well plate, and the absorbance was measured at 405 nm using a microplate reader.

### 4.5. Measurement of Intracellular Tyrosinase Activity

The B16F10 cells were seeded at 8.0 × 10^4^ in 60 mm dishes and incubated for 24 h. Samples were processed in the same way as for the melanin contents. All the medium was removed, the cells were washed twice with cold 1× PBS, and the lysis buffer was added at 200 μL per plate for lysis at 4 °C for 30 min. The cell lysates were harvested and transferred to a 1.5 mL e-tube and centrifuged at 15,000 rpm and −8 °C for 30 min. The separated supernatant was diluted 1:10 with deionized water, and the total protein concentration was quantified using the BCA protein assay kit (Thermo Scientific, Rockford, IL, USA). Then, 80 μL of 2 mg/mL of L-DOPA (in 0.1 M sodium phosphate buffer, pH 6.8) and 20 μL of the quantified protein were diluted and transferred to a 96-well plate. The absorbance was measured at 490 nm using a microplate reader every 30 min at 37 °C, up to a maximum of 1 h and 30 min.

### 4.6. Western Blotting

The B16F10 cells were seeded in 60 mm dishes and treated with varying concentrations of sample and α-MSH (100 nM) to determine the protein level at different time points. For tyrosinase, TRP-1, and TRP-2 expression, cells were seeded at 1.5 × 10^5^ cells/dish and incubated for 48 h following a 24 h stabilization period. MITF and Wnt proteins were expressed in cells seeded at 3.0 × 10^5^ cells/dish and incubated for 24 h after stabilization. To determine the ERK and AKT expression, the same cell density was used, with incubation for 24 h after stabilization, followed by a 4 h treatment with samples. After treatment, the medium was removed, and cells were washed twice with cold 1× PBS. They were then lysed with 200 μL of lysis buffer per well for 30 min at 4 °C. Cell lysates were collected and centrifuged at 15,000 rpm for 30 min at −8 °C. The protein quantification was performed using Pierce™ BCA Protein Assay Kits (Thermo Scientific, Rockford, IL, USA), with the supernatant being diluted and analyzed for absorbance at 562 nm. The proteins were diluted to 30 μg/mL and mixed with a solution of 2× Laemmli sample buffer and 2-mercaptoethanol, followed by incubation at 100 °C for 5 min. For Western blot analysis, 16 μL of each sample was loaded onto an SDS–polyacrylamide gel and subjected to electrophoresis. The proteins were transferred to a PVDF membrane, which was then washed and blocked with appropriate blocking solutions for specific proteins. Primary antibodies were incubated with the membrane overnight at 4 °C, followed by washing and incubation with secondary antibodies. The membranes were washed again, and protein bands were detected using an ECL kit (Thermo Scientific, Rockford, IL, USA) and analyzed with ImageJ software (version 9.4.0.).

### 4.7. Human Skin Patch Test

This study was ethically and scientifically approved by Dermapro’s IRB (No. 1-220777-A-N-01-B-DICN24054) in accordance with the Declaration of Helsinki. Thirty-two women (mean age 43.03 ± 5.17 years) participated, having met the inclusion and exclusion criteria. All participants provided informed consent after being briefed on the study’s purpose, methods, and potential risks. The test substance (20 μL) was applied to a cleaned area on the subjects’ backs for 24 h, followed by skin evaluations at 20 min and 24 h post-application. A dermatologist assessed their skin reactions using the PCPC guidelines. For safety, any severe reactions (+5) were classified as potential allergic responses, and the maximum rating was limited to +4 (Table 2).

$$\mathrm{Response}=\frac{\sum \left(Grade\times No.\;\;of\;Responders\right)}{4\;\left(Maximum\;Grade\right)\times n\;\left(Total\;Subjects\right)}\times 100\times 1/2\;$$

### 4.8. Statistical Analyses

All the experimental results are expressed as the mean and standard deviation (mean ± SD) of at least three replicates. Statistical analysis was performed using WINKS SDA Version 7.0.9 Professional, TexaSoft (Cedar Hill, TX, USA), using *t*-tests to determine the *p*-values: * *p* < 0.05, ** *p* < 0.01, and *** *p* < 0.001.

## Figures and Tables

**Table 1:** Results of the human skin primary irritation test (*n* = 32).

No.	Test Sample	No. of Responses	1st Assessment				2nd Assessment				Reaction Grade (R) *
			+1	+2	+3	+4	+1	+2	+3	+4	
1	6M-4MC (50 μM)	0	0	0	0	0	0	0	0	0	0
2	6M-4MC (100 μM)	0	0	0	0	0	0	0	0	0	0

* None to slight: 0.00 ≤ R < 0.87.

**Table 2:** Grading system for the primary skin irritation test.

Grade	Description of Clinical Observations
+1	Slight erythema
+2	Moderate erythema, possibly with barely perceptible edema at the margin; papules may be present
+3	Moderate erythema, with generalized edema
+4	Severe erythema with severe edema, with or without vesicles
+5	Severe reaction spreading beyond the area of the patch

## Data Availability

The authors confirm that all the data needed to support this study are presented within the article.
